# Efficient Preparation of Poly(allyl diglycol carbonate) (PADC) Nuclear Track Detectors: UV Photopolymerization

**DOI:** 10.3390/polym16131891

**Published:** 2024-07-02

**Authors:** Guangshe Zhang, Li Zhang, Wencheng Gao, Riwei Xu, Kuke Ding

**Affiliations:** 1 Key Laboratory of Carbon Fiber and Functional Polymers, Beijing University of Chemical Technology, Ministry of Education, Beijing 100029, China; zgshe0622@163.com (G.Z.);; 2 Chinese Center for Disease Control and Prevention, Beijing 102206, China

**Keywords:** UV photopolymerization, high efficiency, track detection, PADC

## Abstract

The decay of radon gas in soil and buildings produces alpha radiation, which is the second leading cause of lung cancer in humans. Therefore, by conveniently detecting radon gas in the environment, potential sources of danger can be identified early, and necessary measures can be taken to protect human health. Solid-state nuclear track detectors prepared from polyallyl diglycol carbonate (PADC) resin are the most sensitive detectors for alpha radiation released by radon gas. The traditional method of preparing PADC resin involves free radical thermal polymerization, which suffers from issues such as low polymerization efficiency, long processing time, and the occurrence of defects in the product. In this study, PADC resin was efficiently prepared using a UV initiator. Starting from the polymerization mechanism, experiments were designed using a controlled variable approach, and a rational polymerization apparatus was devised. By comparing the double bond conversion rate, transparency, hardness, and yellowness index of the polymers, the optimal initiator for PADC resin, 2-hydroxy-2-methyl-1-phenyl-1-propanone (1173), was selected. The influence of irradiation intensity, irradiation time, and UV initiator dosage was investigated. The performance of the polymers, including double bond conversion rate, optical properties, dynamic mechanical properties, etching rate, and track detection efficiency, was analyzed. The experimental conditions for preparing PADC resin were optimized: irradiation intensity of 12 mW/cm^2^, irradiation time of 25 min, and UV initiator dosage of 5 parts. The resulting resin polymer had a double bond conversion rate of 93.2% and a track detection efficiency of 0.714.

## 1. Introduction

**Figure 1:**
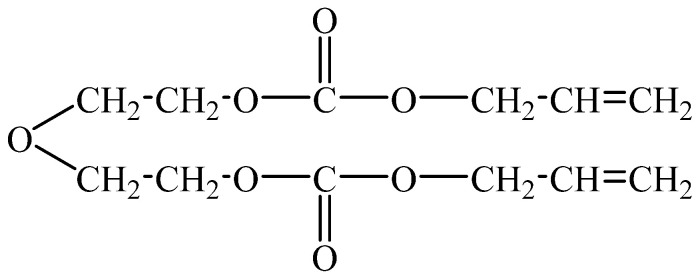
Chemical formula of allyl diglycol carbonate.

In the 1940s, an American company developed a new type of resin material named polyallyl diglycol carbonate (PADC), with its chemical composition being allyl diglycol carbonate (ADC). It was marketed under the brand name Columbia Resin 39, abbreviated as CR39 [1]. The chemical formula of this resin monomer can be represented as (CH_2_=CHCH_2_OCOOCH_2_CH_2_)_2_O, with its chemical structure depicted in Figure 1. For a long time thereafter, PADC resin was utilized in optical applications. It was not until 1978 that B. G. Cartwright and E. K. Shirk discovered another significant application for PADC resin [2]. This resin could be used to detect nuclear tracks, serving as a solid-state nuclear track detector. Subsequently, an increasing number of researchers began investigating the track detection performance of PADC resin. Compared to other materials, CR39 resin stands as the most useful solid-state nuclear track detector material presently available [3]. According to the United Nations Environment Programme (UNEP), humans are exposed to 80% of natural radiation sources and 20% of artificial radiation sources. Within natural radiation sources, radon gases (^219^Rn, ^220^Rn, ^222^Rn) and their progenies account for 42% of this dose. Hence, detecting radiation sources in the environment is crucial for human safety. CR39 resin is presently recognized as the most sensitive material to α-radiation, and detectors made from it are widely used to measure ^222^Rn in dwellings, offices, underground mines, caves, water, and ventilation systems [4].

The structures [5] and properties of PADC resin are determined by the polymerization and curing process of the monomer allyl diglycol carbonate [6]. Typically, the curing of PADC involves heat treatment using organic peroxides such as diisopropyl peroxodicarbonate (IPP), cyclohexyl peroxodicarbonate (CHPC), or benzoyl peroxide (BPO). These organic peroxides decompose into free radicals, initiating the polymerization of allyl groups.

Benzoyl peroxide (BPO) is initially used as the initiator, with polymerization controlled at around 70 °C [1]. The preparation of lenses required approximately 70 h, involving higher reaction temperatures and longer reaction times. Using IPP and CHPC offers the advantage of not requiring excessively high polymerization temperatures compared to those needed when using BPO. However, IPP and CHPC need lower storage temperatures and may pose risks during transportation [7,8].

When initiating the polymerization of ADC using peroxides, the process involves three stages [9,10]: initiation, propagation, and termination. Peroxides undergo thermal decomposition, generating free radicals that bind to the allyl groups of ADC to form allyl radicals. Subsequently, the allyl radicals on ADC undergo propagation, forming polyallyl chains linked by ethylene glycol carbonate units, creating a tightly cross-linked three-dimensional polymer network. Ultimately, termination of polyallyl chains occurs.

Conventionally, in free radical polymerization, termination often involves biradical coupling or disproportionation of two radicals to deactivate each other. However, for the polymerization of allyl monomers, the most crucial termination mechanism involves chain transfer, wherein growing alkyl radicals annihilate by abstracting a hydrogen atom from the monomer, converting allyl radicals into relatively stable allyl radicals [11]. Free radicals produced during the ADC polymerization process can survive for at least six months within the CR-39 polymer. The most likely radicals to endure are the resonantly stabilized allyl radicals [9,12].

During the polymerization of ADC monomers, the overall rate of polymerization is usually controlled by the initiation step. Peroxide initiators, upon thermal decomposition, initiate free radical polymerization. Achieving the necessary concentration of free radicals often requires maintaining higher temperatures, but excessively high temperatures can hinder heat dissipation during the exothermic process of allyl radical polymerization. This thermal imbalance within the system may result in overheating and uneven internal stresses in the polymer, leading to internal defects [13]. One method to address this issue, proposed in 1955, involves a gradient temperature polymerization process, typically prolonged and reducing resin preparation efficiency, elongating experimental processes [1,14].

Another approach to addressing this issue is by utilizing UV light to initiate the preparation of PADC. Compared to thermal polymerization and redox polymerization, photopolymerization offers numerous advantages. In thermal and redox polymerizations, the reaction system generates active centers through heating, whereas photopolymerization employs highly efficient photoinitiators, and spatial and temporal control of polymerization is achieved by controlling the type and intensity of the initiating light. Photopolymerization requires less energy for curing compared to thermal polymerization. Its curing speed is remarkably rapid, allowing for the processing of a larger quantity of thin sheets and coatings in a shorter period. Additionally, photopolymerization systems are more compact than thermal curing systems and can be operated at room temperature. All these features of photopolymerization contribute to enhanced polymerization efficiency and reduced costs [15,16]. Stejny et al. [10] developed UV-initiated ADC monomers utilizing benzoin ethyl ether (BEE) and 2,2-dimethoxy-2-phenylacetophenone (DMPA) to prepare PADC polymers for track detection. However, their study has limitations such as relatively high polymerization temperature, lengthy reaction time, and uneven surfaces of the resulting sheets.

Therefore, this study aimed to improve upon prior work by utilizing the 1173 photoinitiator. A novel polymerization setup is designed, enhancing polymerization efficiency by enabling the curing of ADC monomers within an hour at room temperature, achieving over 90% double bond conversion within the polymer. This advancement allowed for a broader exploration of UV-initiated PADC resin in track detection studies. UV curing allows significant flexibility in curing conditions without the need for specific temperatures and curing times to avoid overheating. This study provides new insights into enhancing the production efficiency of PADC track sheets.

## 2. Materials and Methods

### 2.1. Materials

The ADC monomer was purchased from Heowns. Six types of UV initiators were tested, namely, 2-hydroxy-2-methylpropiophenone (1173) and 2-methyl-4′-(methylthio)-2-morpholinopropiophenone (907) from Aladdin (Shanghai, China), diphenyl (2,4,6-trimethylbenzoyl) phosphine oxide (TPO) from Shanghai Yuanye Bio-Technology (Shanghai, China), 2-hydroxy-4′-(2-hydroxyethoxy)-2-methylpropiophenone (2959) from Macklin (Shanghai, China), phenylbis(2,4,6-trimethylbenzoyl)phosphine oxide (819) from Bide Pharmatech (Shanghai, China), and 1-hydroxycyclohexyl phenyl ketone (184) from Beijing Chemical Works (Beijing, China). All reagents are of analytical grade and used as received.

### 2.2. Curing Procedure

Initially, a specific amount of monomer was carefully weighed and then a precise quantity of initiator, such as 5 phr (parts per hundred of the ADC), was also weighed. Subsequently, both substances were deposited into a glass vial. After initial agitation to facilitate blending, the lid of the glass vial was tightly secured. The sealed glass vial was then submersed into a water bath and heated to 60 °C to completely dissolve the initiator into the monomer. This process continued until total dissolution was achieved. The resultant solution was transferred into a small beaker and homogenized using a homogenizer (HR-500DG, Shanghai HuXin Industrial Co., LTD, Shanghai, China, operating at 6000 r/min). Finally, the mixture was placed into a vacuum oven under vacuum conditions to evacuate any remaining gases (room temperature, 0.09 MPa, 30 min).

After carefully extracting the homogenized mixture of monomer and initiator from the beaker, we cautiously injected it into the mold, ensuring no air bubbles were introduced as they might compromise the quality of the final product. Once the monomer was successfully transferred, we moved the mold onto a turntable rotating at a speed of 1 rpm (1 r/min). We employed two LED-UV curing lamps (50w, LEDDIAN Lighting Company, Dongguan, China) for a 20 min exposure duration—one positioned in front of the mold and the other at a 90 degree angle to the side of the mold—to initiate the polymerization reaction. We maintained a distance of 10 cm between the UV lamp and the mold surface, using a UV lamp with a wavelength of 365 nm and an intensity of approximately 12 mW/cm^2^, at an ambient temperature of around 20 °C.

**Figure 2:**
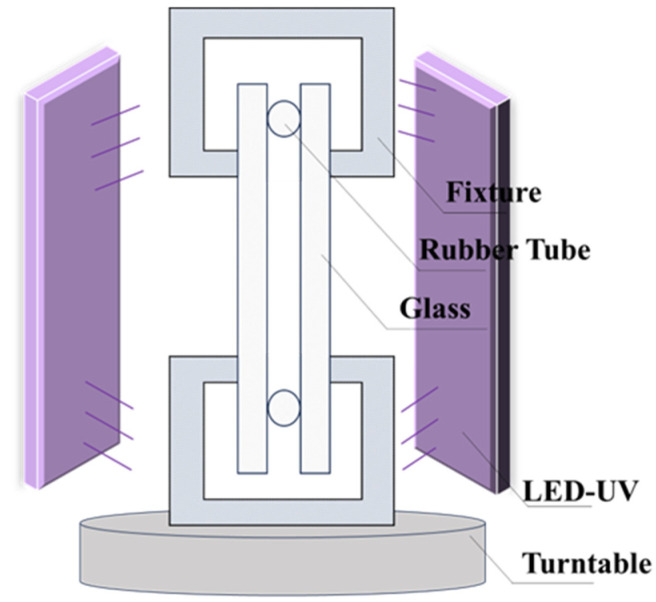
Schematic diagram of polymerization apparatus.

The mold used in this experiment consisted of high borosilicate glass (200 mm × 200 mm × 5 mm), rubber strips, and fixtures, as depicted in the Figure 2. It is important to note that the edges of the rubber strips should not be too close to the edges of the glass plates. Additionally, the appropriate tightness of the fixtures is crucial. Excessive looseness might result in monomer leakage during polymerization, while excessive tightness could lead to excessive shrinkage during the polymerization process (due to inherent volume reduction in monomer polymerization), resulting in patterns on the product’s surface that could affect its properties and applications. Furthermore, it is essential to maintain consistent positions and levels of tightness of the fixtures to control variables and prevent interference with experimental results [17].

### 2.3. Degree of Polymerization Reaction

The degree of polymerization reaction, also known as conversion, is expressed as the ratio between the amount of allyl groups consumed during the polymerization reaction and the initial amount of allyl groups present at the start of polymerization. The Fourier transform infrared spectroscopy (FTIR) method is utilized to determine this conversion rate. The Nicolet-IS5 Fourier Transform Infrared Spectrometer (Thermo Fisher Scientific Inc., Waltham, MA, USA) was employed for testing purposes. The liquid samples were prepared using the KBr pellet method, while the solid gel-like samples were analyzed using ATR (Attenuated Total Reflection). The data obtained from measuring the sample surface using the ATR method are highly reliable, with a detailed analysis provided in the ESI (see Figure S1) via OMNIC 8.0. The scanning range for both methodologies was set between 500 and 4000 cm^−1^, with a total of 128 scans per sample.

According to Lambert–Beer’s law, when the molar absorptivity of a substance and the thickness of the absorption layer remain constant, absorbance is directly proportional to concentration. During the progression of the polymerization reaction, the concentration of carbon–carbon double bonds (C=C) decreases. Thus, the concentration of C=C bonds can be used to indicate the conversion rate of the monomer.

**Figure 3:**
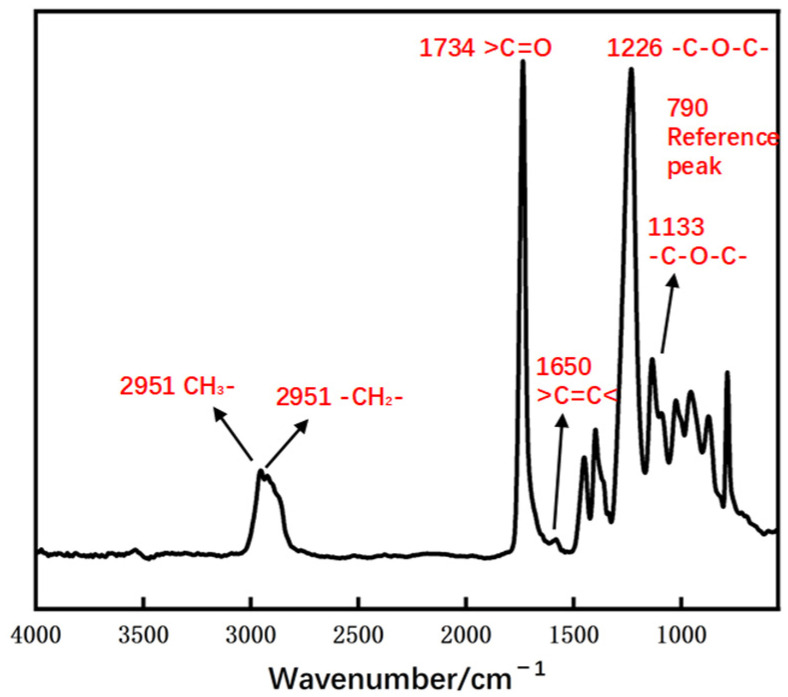
Absorption bands of various chemical bonds in the FTIR spectrum of PADC [3].

In the FTIR spectrum, the absorption peak at 1649 cm^−1^ corresponds to the C=C bonds, while the absorption peak at 790 cm^−1^ for the carbonate group is selected as the reference peak [18]. Figure 3 shows the absorption bands of various chemical bonds in the FTIR spectrum of PADC. Let A denote the ratio of the peak absorbance of the C=C bonds to the absorbance of the reference peak for each sample. When the conversion rate reaches 100%, A equals 0. The conversion rate x is calculated using Equation (1).

The formula for determining the conversion rate x of PADC monomers using FTIR spectroscopy is as follows:

(1) $$x=[(A_{0}-A)/A_{0}]\times 100\%$$

The variables in the equation are defined as follows: x represents the conversion rate, and A0 is the ratio of the peak absorbance of carbon–carbon double bonds to the absorbance of the reference peak when the conversion rate is zero (pure monomer).

### 2.4. Density and Shore D

For solid samples, density is measured using the hydrostatic weighing method. This method involves suspending the grounded sample piece in a deaerated, non-expansive liquid for measurement. The liquid used must exhibit good wetting properties with the sample and should not induce any expansion in the sample. In this study, deionized water was chosen as the non-expansive liquid [19]. However, the density is also influenced to a slight extent by the length of the polyallyl chains. This is due to the end units of the chains having a higher free volume compared to the repeating units within the chain, thereby resulting in an increase in density with the chain’s length [10].

The Shore hardness tester model used in this experiment was LX-D-2 (Wenzhou Weidu Electronics Co., Ltd., Wenzhou, China). The D-type is suitable for general hard rubber, resins, acrylics, glass, thermoplastic rubber, printing plates, fibers, and similar materials. The density of the polymer was measured at room temperature using a densitometer (DB-300G, Shanghai Jingqi Instrument Co., Ltd., Shanghai, China).

### 2.5. Irradiation and Etching

Etching is performed using an etching solution. The entire track etch sheet was laser-cut into dimensions of 3 cm × 5 cm, cleaned of surface dust with deionized water, dried, and then placed parallel to a mixed source of isotopes ^238^U + ^226^Ra + ^232^Th at a distance of 1 cm for 1 h of irradiation [4]. Following the irradiation, the sample is rinsed to remove surface dust, then etched vertically in a 6.25 M/L NaOH solution for 6 h at a temperature of 70 °C [20,21].

After the etching process, the etched track etch sheets were observed under an optical microscope (ML31, Guangzhou Mingmei photoelectric technology Co., Ltd., Guangzhou, China), and photographs were taken for examination.

The overall etching rate (V_b_) is determined through a weight measurement method, utilizing the following formula [22]:

(2) $$V_{b}=\frac{△m}{2A\rho t}$$

where Δm represents the loss in polymer weight during etching time ‘t’, A denotes the surface area of the sheet, and ρ stands for the density of the material [23].

The detection efficiency of the trace element detector produced by the polymer is an important performance parameter. Nicholas Tsoulfanidis [24] defined detection efficiency as the ratio of the net number of particles detected by the detector per unit time to the number of particles directed towards the detector per unit time. The activity of the standard radiation source is known, and the track detection efficiency can be obtained by measuring the track density. In this study, the standard radioactive source used is a mixed source of isotopes ^238^U + ^226^Ra + ^232^Th, with an α-radioactive specific activity of 168 ± 1.2 α-decays/cm^2^·min.

### 2.6. The Determination of Transmittance

A UV-visible spectrophotometer (TU-1810, Beijing PUXI General Instrument Co. LTD, Beijing, China) is a commonly used experimental instrument for analyzing the absorption of UV-visible light by molecules. In the testing process, samples are placed in a sample cell holder, and the scanning mode is set to spectrum scan, with a fast scanning speed and a wavelength range of 400 to 800 nm. These data are essential for studying the optical properties and chemical characteristics of substances.

### 2.7. Dynamic Mechanical Analysis

The polymer sheets were tested using the TA DMA Q800 (Thermo Fisher Scientific Inc., Waltham, MA, USA) instrument in the single cantilever mode. Temperature/time scans were conducted at a test frequency of 1 Hz with a dynamic temperature ramp of 5 °C/min [25]. Typically, the glass transition temperature (*T_g_*) of the polymer appears at the inflection point of the Tan curve obtained from Dynamic Mechanical Analysis (DMA).

## 3. Results and Discussion

### 3.1. The Influence of Initiators on Polymer Properties

**Figure 4:**
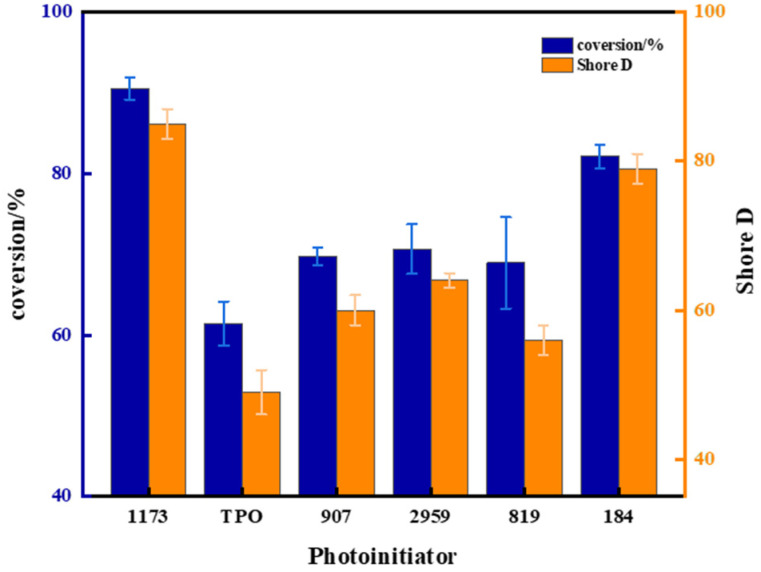
Conversion rate for monomers and Shore D of polymers initiated by different photoinitiators.

**Figure 5:**
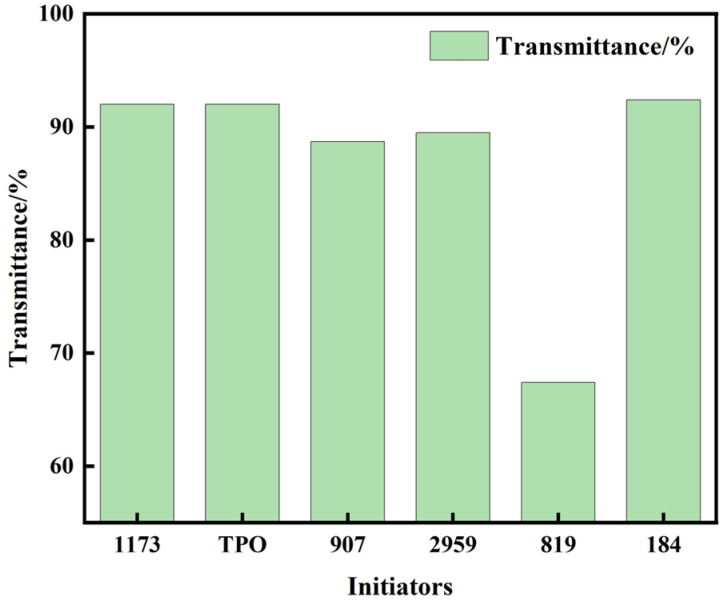
The transmittance of polymers initiated by different photoinitiators at 550 nm.

**Figure 6:**
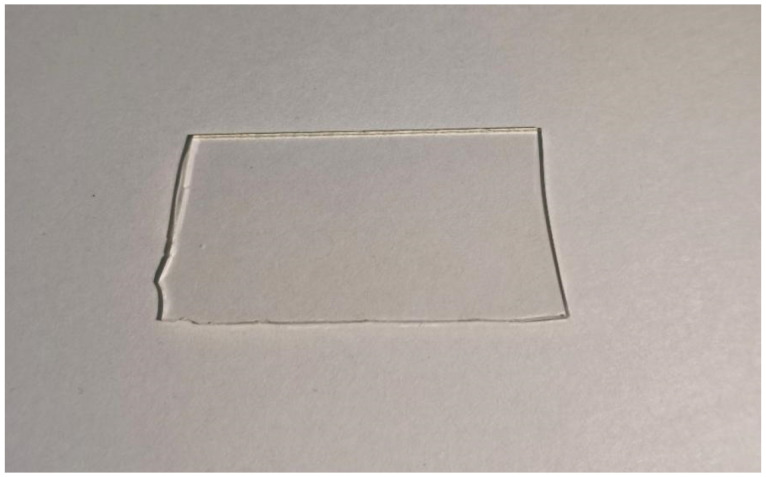
PADC prepared using photoinitiator 1173.

In order to find the suitable photoinitiator, ADC monomer polymerization was induced using various initiators. These included α-hydroxy ketones: 1173, 184, 2959; α-amino ketone: 907; and acylphosphine oxide compounds: TPO, 819. The addition of different photoinitiators under the same conditions of UV irradiation leads to significant differences in the conversion rate of the resulting polymers. The experimental results are shown in Figure 4. The addition of TPO resulted in the lowest double bond conversion rate, merely reaching 61.4%. Double bond conversion rates initiated by 907, 2959, and 819 photoinitiators were quite similar, measuring at 69.7%, 70.6%, and 66.7%, respectively. The double bond conversion rate achieved with the addition of photoinitiator 184 was relatively higher at 82.1%, although still exhibiting some difference compared to photoinitiator 1173. The use of photoinitiator 1173 yielded the highest double bond conversion rate at 90.6%. Remarkably, the trend observed in polymer hardness aligned closely with the corresponding double bond conversion rates. Polymers initiated with 1173 demonstrated the highest shore hardness at 85 HD, while those initiated with 819 displayed the lowest hardness at 56 HD. Analyzing the outcomes revealed that, among the photoinitiators tested for ADC, 1173 proved to be the most efficient. From the analysis of Figure 5, it can be observed that for materials obtained using 1173 as the photoinitiator, a high transparency of 92% was observed at a wavelength of 550 nm, indicating excellent optical properties of the polymer. Figure 6 shows PADC prepared using photoinitiator 1173.

### 3.2. Light Exposure Duration and Polymer Properties

Subsequently, the study focused on the impact of exposure time on the properties of the polymer. Monomers contain allyl double bonds that can undergo free radical chain polymerization under UV irradiation. Double bond conversion was calculated according to FTIR of polymer (see Figure S2).

**Figure 7:**
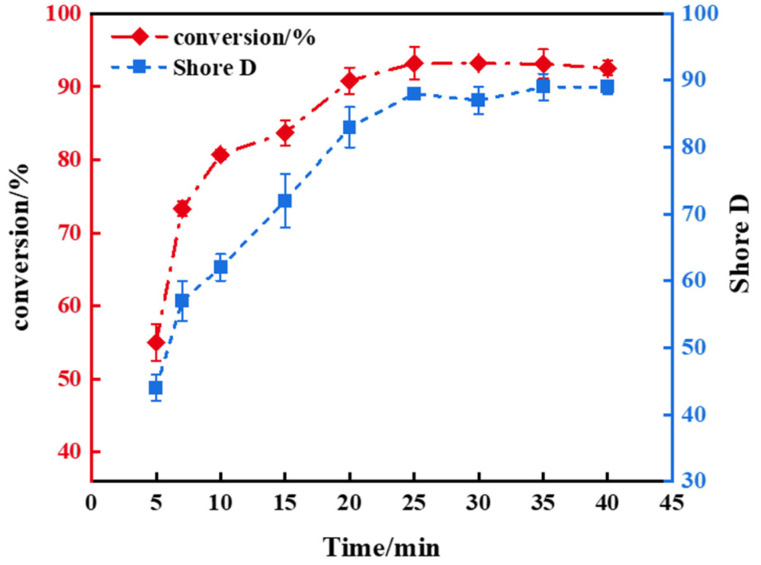
The dependence of double bond conversion rate and Shore D on different UV exposure times for the polymer with a 5 phr addition of 1173.

Figure 7 illustrates the dependence of product conversion rates and hardness on UV exposure time. The results indicate that both the conversion rate and hardness increase with extended UV exposure time. However, after 25 min, the polymer reached its maximum conversion rate of 93.2% and a hardness of 88 HD, showing no further increase. During curing, UV light exposure initiates the rapid decomposition of initiators into numerous free radicals. Hence, during the initial phase of curing, the initiator concentration should be relatively high. Subsequently, free radicals engage in chain polymerization within the system, causing rapid monomer aggregation and a gelation phenomenon, escalating the system’s viscosity. This elevated viscosity restricts the mobility of unreacted free radicals, preventing their continued reactivity. This indicates that the double bond conversion rate of the polymer has reached its limit at this point.

**Figure 8:**
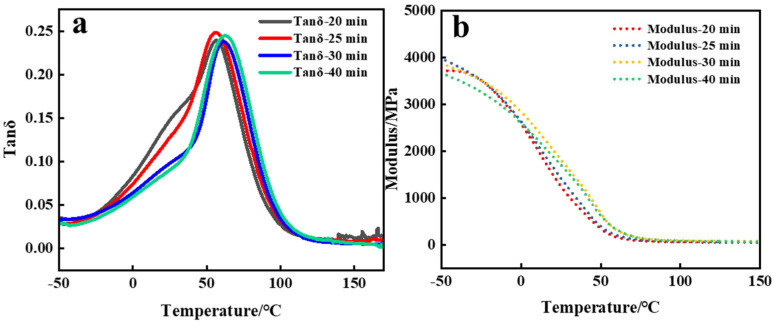
(**a**) Tanδ and (**b**) modulus curves of polymers polymerized under different irradiation times with the addition of 5 phr 1173.

As demonstrated in Figure 8, the DMA curves for various polymerization durations exhibited the peak value of tanδ appeared at 61.94 °C, signifying the *T_g_* of the corresponding polymers in light-induced ADC polymerization. With increasing exposure time to light, the *T_g_* of the polymer was also elevated. Cross-linking augmented the hardness and rigidity of the polymer while reducing its elongation capability. In other words, as the exposure duration to light in the polymerization system increased, the density of cross-linking also increased. As a result, the polymer material exhibited greater rigidity; lower chain mobility; and subsequently, a higher *T_g_*. It is worth noting that although *T_g_* was higher in the former, the difference was not large. Moreover, between −50 °C and −60 °C, the rate of decrease in the storage modulus for photo-initiated polymers was faster than that of thermally initiated polymers, possibly due to their lower cross-linking density, facilitating smoother chain segment sliding.

### 3.3. The Dosage of Photoinitiators and the Performance of the Product

**Figure 9:**
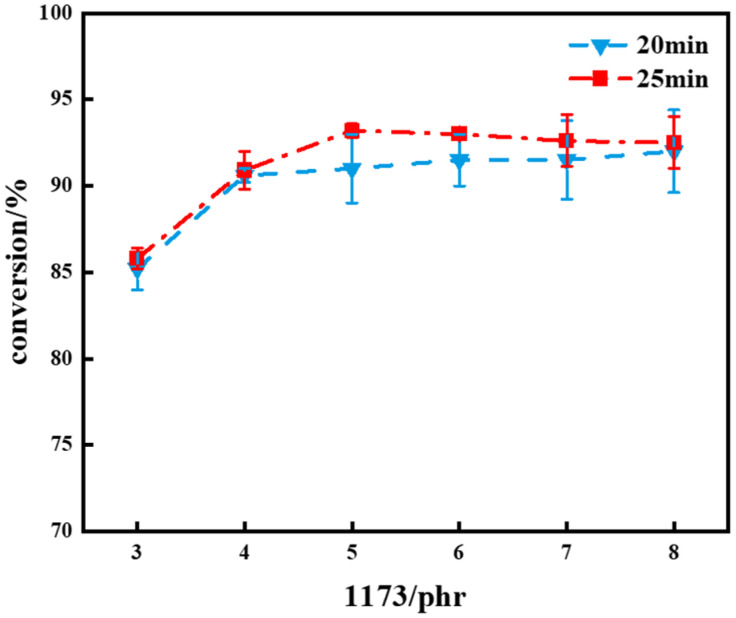
A comparison chart of the double bond conversion rates of polymers with varying amounts of 1173 under UV exposure times of 20 min and 25 min.

Afterward, the investigation delved into the effect of initiator quantity on the polymer. Figure 9 illustrates a comparison of the double bond conversion rates with different additions of 1173 photoinitiator under various UV light exposure times. The analysis revealed that the conversion rate of the polymer under 25 min of light exposure was higher compared to that under 20 min. At 20 min of UV exposure, the polymer conversion rate stabilized when the photoinitiator dosage exceeded 4 phr. At 25 min of light exposure, the polymer’s conversion rate peaked with the addition of 5 phr 1173, and further increments in the initiator addition did not contribute to a higher conversion rate.

From the data, it can be analyzed that with an increase in initiator quantity, the C=C bond in the polymer reacted more completely (see Figure S3). However, when the initiator quantity exceeded a certain threshold, the conversion rate no longer increased but instead declined. Further addition of the initiator did not contribute to an increase in the conversion rate, showing diminishing returns. The termination of free radical recombination or dismutation relies on the square of the free radical concentration. Consequently, these termination processes become notably prominent with higher free radical concentrations, resulting in shorter lengths of polyallyl chains and a decrease in the efficiency of the initiator [10]. Therefore, an excess of the added photoinitiator leads to a reduction in the polymer conversion rate. At a 25 min exposure to light, a turning point in the trend of hardness and density of the polymer was observed with the addition of 5 phr 1173. This indicates that under this condition, the polymer reached its maximum hardness and density.

**Figure 10:**
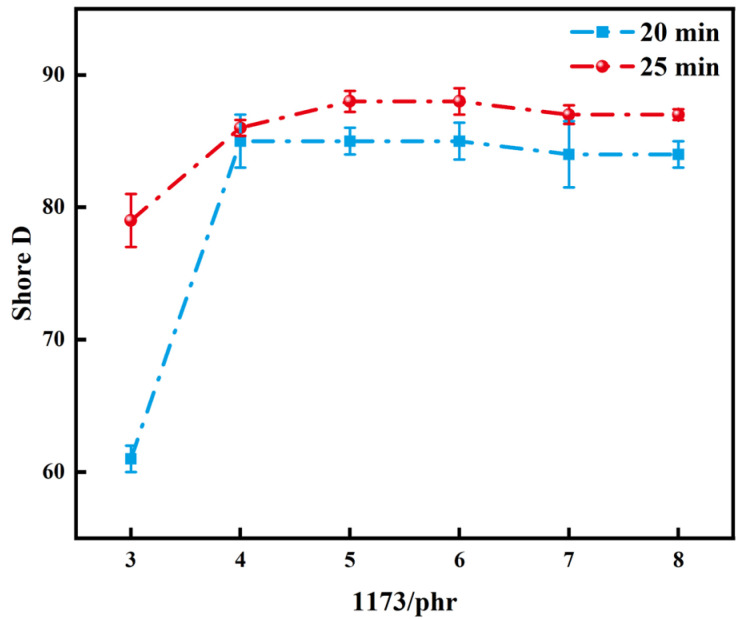
Shore D of polymers with different amounts of 1173 added under light exposure times of 20 min and 25 min.

**Figure 11:**
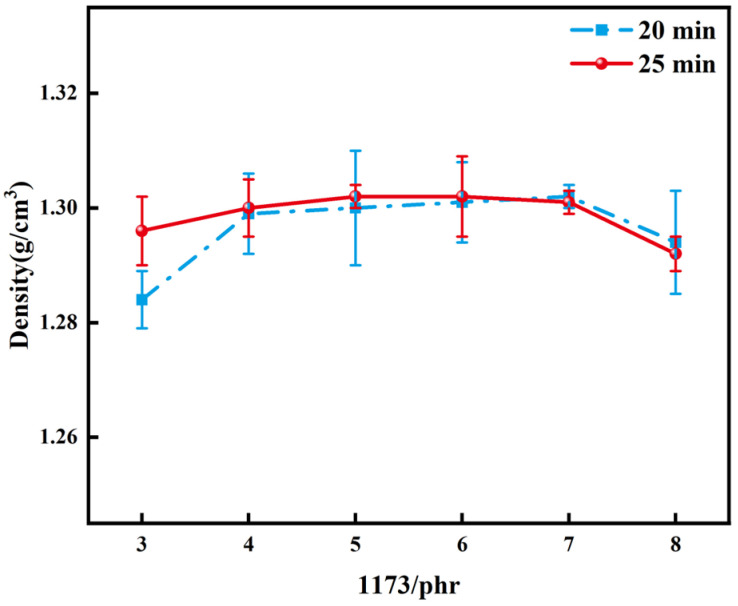
Density of polymers with different amounts of 1173 added under light exposure times of 20 min and 25 min.

Figure 10 and Figure 11 demonstrate the changes in Shore D and density of polymers, respectively, when different amounts of initiator were added under light exposure times of 20 min and 25 min. Analyzing both graphs revealed a gradual increase in polymer hardness and density with increasing initiator addition. However, the initiator required to achieve maximum hardness and density of polymers under different light exposure times varied. For a light exposure time of 20 min, the polymer’s maximum hardness was achieved with the addition of 4 phr of 1173, with little change in density beyond this value. Conversely, a light exposure time of 25 min resulted in the resin’s maximum hardness and density with the addition of 5 phr of 1173. Taken together, the addition of 5 phr of 1173 under a light exposure time of 25 min appeared to be the most suitable experimental condition.

**Figure 12:**
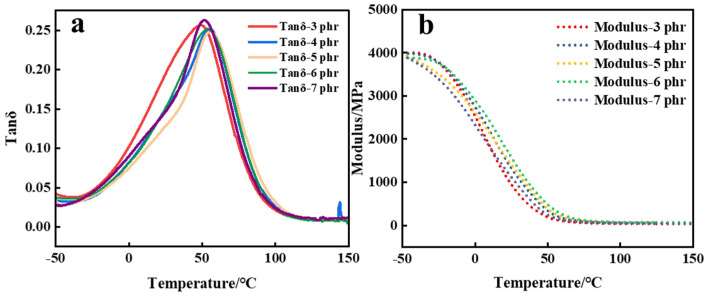
(**a**) Tanδ and (**b**) modulus curves of polymers with different concentrations of 1173.

The PADC resins with different quantities of 1173 were subjected to DMA testing, and the results are depicted in Figure 12. From the DMA graph, it is evident that the polymer’s *T_g_* varied with different amounts of photoinitiator. Notably, the highest *T_g_* of the PADC was observed when 5 phr 1173 was used. It was evident that an excessive amount of 1173 added under the same light exposure time led to a reduction in the polymer’s *T_g_* and storage modulus. This aligns with the declining trends observed in Figure 10 regarding the decrease in conversion rate and hardness. The increase in the conversion rate of the ene-functional groups with an increase in the initiator content led to an elevation in *T_g_*. However, this effect diminished due to a reduction in the length of the polyallyl chains. At high initiator concentrations [26], the increase in initiator addition has a minimal effect on double bond conversion rates. However, the predominant factor causing the decline in *T_g_* was found to be the shortening of the chains, initiating a decrease in *T_g_*. When the initiator amount was 5 phr, the *T_g_* of the polymer was the largest, indicating that the cross-linking density of the polymer was the largest.

### 3.4. Track Detection

**Figure 13:**
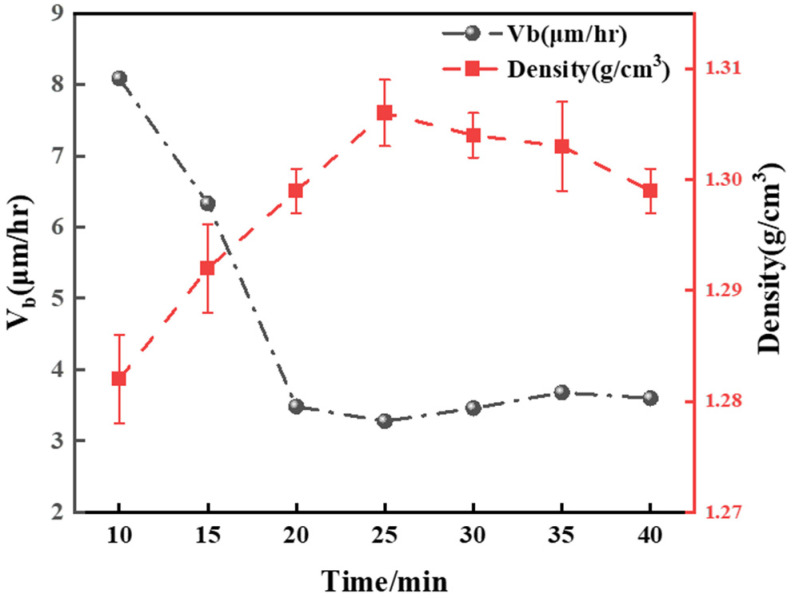
The overall etching rate (V_b_) of polymers after adding 5 phr of 1173 and exposing to UV light for 25 min, as a function of density.

As shown in Figure 13, the changes in the bulk etch rate and density of the polymer after different UV exposure times are depicted for a 5 phr addition of the photoinitiator 1173. Analysis of the curves reveals that as the PADC density reached its peak at 1.306 g/cm^3^, the bulk etch rate concurrently reached its minimum at 3.27 μm/h. This occurred after a UV exposure duration of 25 min.

**Figure 14:**
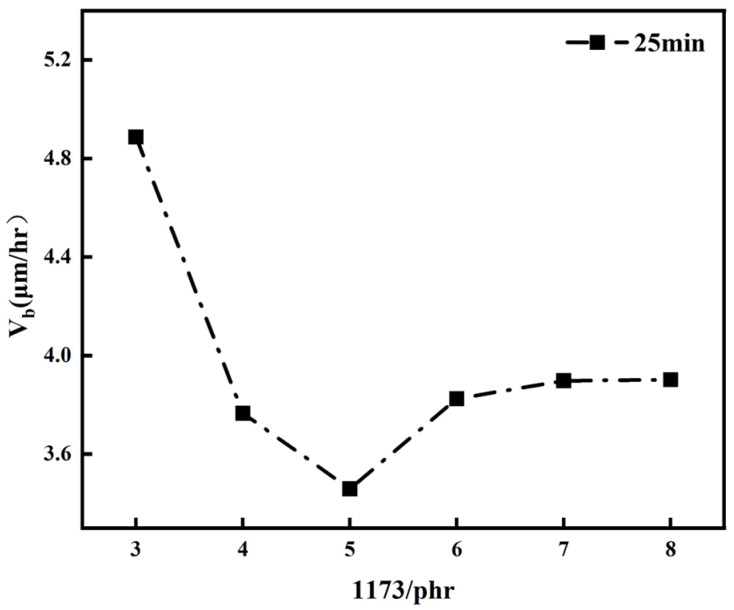
The overall etching rate (V_b_) of polymers at 20 min and 25 min of UV exposure, as a function of the amount of 1173 added.

The dependency of V_b_ on the addition of the photosensitive initiator 1173 is illustrated in Figure 14 at UV exposure times of 25 min. From the curves depicted in the graph, it can be analyzed that at lower initiator concentrations, the overall etching rate of PADC decreased with an increase in the initiator quantity, reaching a minimum at a 5 phr addition. Beyond this amount, the V_b_ of PADC also increased, inversely proportional to the conversion rate and following the same trend as hardness and density variations. The bulk etching rate is a crucial parameter affecting the detection efficiency of track detectors. By studying the bulk etching rate of different polymers and the variations among them, it can be deduced that the trend of the bulk etch rate in relation to the polymer density exhibited an inverse relationship over the duration of UV exposure, aligning with the trend observed in the conversion rate. In polymer systems with higher radical concentrations, the chain length of polyallyl chains became shorter due to chain termination. With high chain-end mobility, an increase in free volume occurred, subsequently leading to a reduction in polymer density, hardness, and the overall etch rate V_b_. At 25 min of UV exposure, the optimal overall etch rate for the polymer occurred with the addition of 5 phr of 1173.

**Figure 15:**
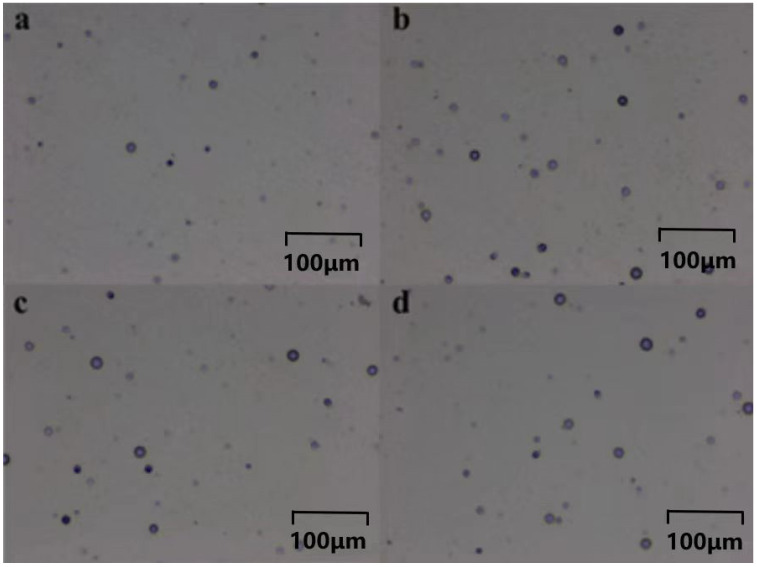
Under the addition of 5 phr 1173, the polymer’s tracks were observed at varying exposure durations of (**a**) 20 min, (**b**) 25 min, (**c**) 30 min, and (**d**) 40 min.

Figure 15a–d displays the etched particle tracks in PADC sheets exposed to radiation after curing at room temperature for 20 min, 25 min, 30 min, and 35 min with the addition of 5 phr 1173. Both fission fragment and α particle tracks were observed, showing strong resemblance to the tracks found in conventional CR-39 cured materials. The detection efficiency of plastic is a critical performance parameter. According to the detection efficiency formula, the track detection efficiency of the polymer was calculated to be between 0.398 and 0.714. The highest track detection efficiency was observed for a 25 min exposure time, at 0.714.

**Figure 16:**
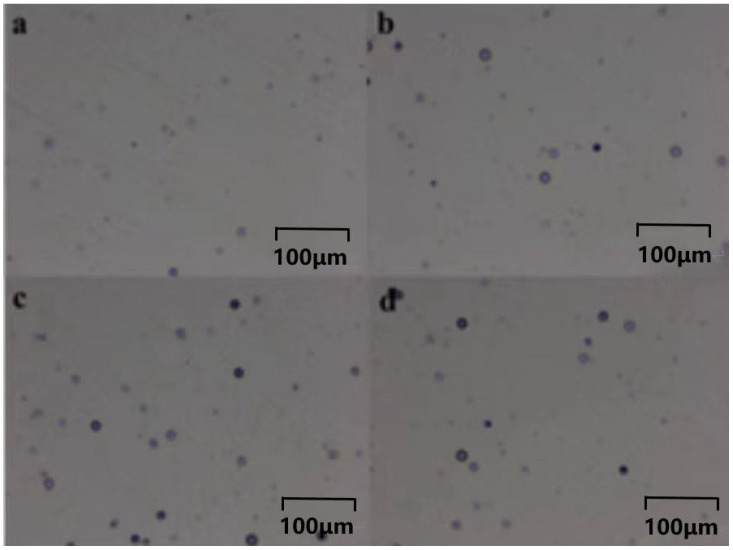
The traces of polymers with different amounts of 1173 added, namely, (**a**) 3 phr 1173, (**b**) 4 phr 1173, (**c**) 5 phr 1173, and (**d**) 6 phr 1173, after exposure to UV light for 25 min.

Figure 16a–d illustrates the effects of different additions of 1173 in polymer track detection after a 25 min curing period. Both fission fragment and α particle tracks were observed, bearing striking resemblance to the tracks found in conventionally cured CR-39 materials. Notably, the detection efficiency of the tracks from the polymer containing 5 phr 1173 exhibited the highest value at 0.714. Overall, it is evident that the addition of 5 phr of 1173 photoinitiator under a 25 min curing condition yielded the optimal results.

## 4. Conclusions

The main conclusions of this study involve the comparative analysis of various UV-sensitive initiators for initiating ADC monomers and selecting more suitable initiators, along with their dosage and conditions. The UV-initiated curing of monomers using UV initiators yielded PADC polymers suitable for track detection. One advantage of this method is that the curing rate is controlled by the amount of photo-initiator added and the conditions of UV radiation, whereas traditional peroxide curing requires temperature control. This allows researchers more flexibility in selecting curing conditions, thus optimizing the detection performance of track detectors. Moreover, it eliminates the need to control temperature during curing cycles to prevent thermal runaway, offering new support for research on the application of photopolymerization in solid-state nuclear track detectors.

## Data Availability

The original contributions presented in the study are included in the article/supplementary material, further inquiries can be directed to the corresponding author.

## Supplementary Materials

The following supporting information can be downloaded at: https://www.mdpi.com/article/10.3390/polym16131891/s1, Figure S1: Comparison of the Surface and Cross-Section of PADC Using the ATR Method. Figure S2; FTIR of polymers with different UV exposure times. Figure S3: FTIR spectra of polymers with different amounts of 1173 added under 25 min of light exposure.
