# Supplementary material for: Efficient Preparation of Poly(allyl diglycol carbonate) (PADC) Nuclear Track Detectors: UV Photopolymerization
Source: Polymers (Basel). 2024 Jul 2;16(13):1891. doi: 10.3390/polym16131891 (PMC11243999; doi:10.3390/polym16131891)
Supplement: Supplementary file 1 [file polymers-16-01891-s001.zip › polymers-3042949-supplementary.pdf]

Supporting information

Efficient Preparation of Poly(allyl diglycol carbonate) (PADC) Nuclear Track Detectors: UV Photopolymerization

Guangshe Zhang [1], Li Zhang [2], Wencheng Gao [1], Riwei Xu [1,*] and Kuke Ding [2,*]

1. Materials and Methods

Details of materials and methods were presented in the context of article.

2. Results and Discussion for FTIR of PADC

FTIR tests were conducted on both the surface and cross-section of PADC using the ATR method. The results are shown in Figure S1. The ATR measurements of the PADC surface and cross-section exhibit relatively consistent peaks, and the calculated double bond conversion rates differ by no more than 2.2%, which is within a reasonable range. Therefore, ATR can be used to measure the double bond conversion rate.

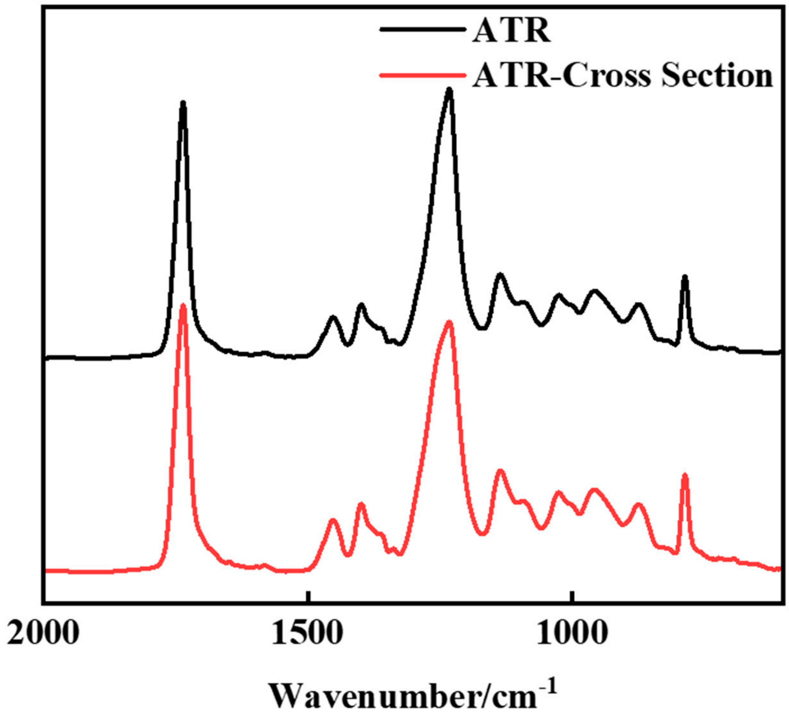

Figure S1. Comparison of the Surface and Cross-Section of PADC Using the ATR Method.

Figure S2 depicts the infrared spectra showing the double bond conversion rates under different UV irradiation times. The extent of the absorption peak of the carbon-carbon double bond at 1650 cm^{-1} indicates that as the UV exposure time lengthens, the C=C bonds within the polymer undergo more complete reactions, resulting in higher corresponding conversion rates.

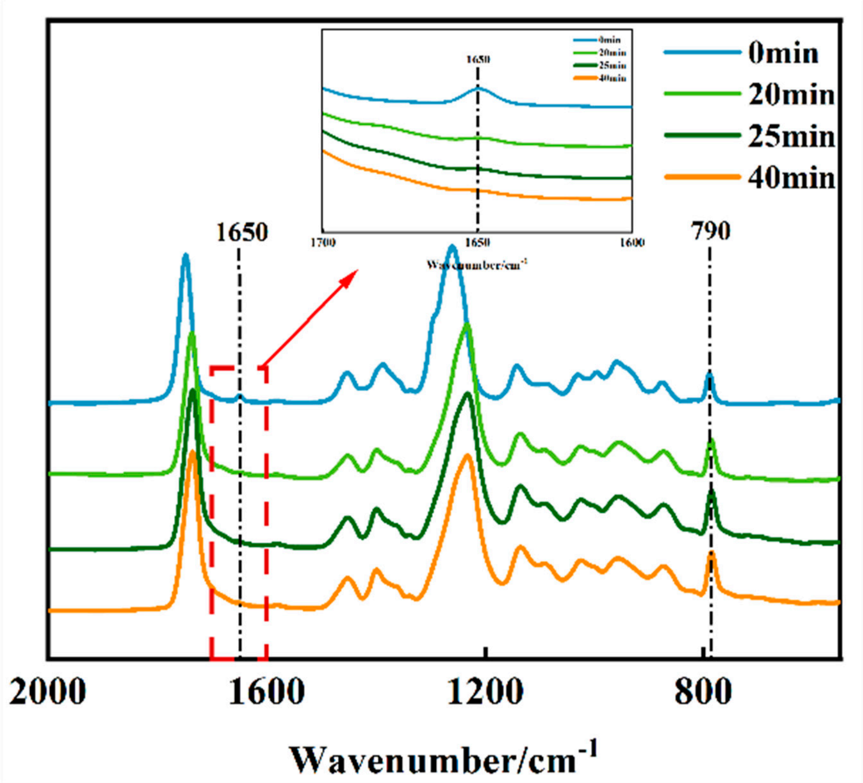

Figure S2. FTIR of polymers with different UV exposure times

Figure S3 shows the infrared spectra of polymers with different addition of initiators added under 25min illumination time. The absorption peak at 1650 cm^{-1} gradually decreases with increasing initiator concentration.

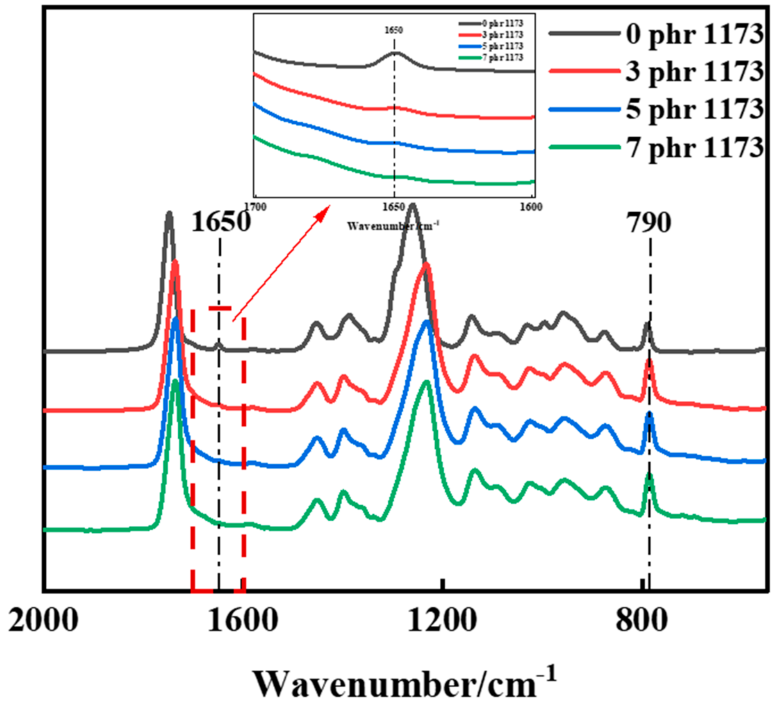

Figure S3. FTIR spectra of polymers with different amounts of 1173 added under 25 min of light exposure.
